# The Role of IL-37 and IL-38 in Colorectal Cancer

**DOI:** 10.3389/fmed.2022.811025

**Published:** 2022-02-02

**Authors:** Jie Dang, Zhiyun He, Xiang Cui, Jingchun Fan, David J. Hambly, Brett D. Hambly, Xun Li, Shisan Bao

**Affiliations:** ^1^Child and Adolescent Health Management Center, Lanzhou University Second Hospital, Lanzhou, China; ^2^Department of General Surgery, Lanzhou University First Hospital, Lanzhou, China; ^3^Department of General Surgery, Lanzhou University Second Hospital, Lanzhou, China; ^4^Department of Epidemiology and Evidence-Based Medicine, School of Public Health, Centre for Evidence-Based Medicine, Gansu University of Chinese Medicine, Lanzhou, China; ^5^Resident Training Program, Gold Coast University Hospital, Southport, QLD, Australia; ^6^Centre for Healthy Futures, Torrens University Australia, Sydney, NSW, Australia

**Keywords:** IL-37, IL-38, colorectal cancer, TAM, TAN

## Abstract

Colorectal cancer (CRC) is a major killer. Dysregulation of IL-37 and IL-38, both anti-inflammatory cytokines, is observed in auto-immune diseases. The precise regulatory mechanisms of IL-37/IL-38 during the development of CRC remains unclear, but chronic intestinal inflammation is involved in the carcinogenesis of CRC. Constitutive production of colonic IL-37 and IL-38 is substantially reduced in CRC, consistent with an inverse correlation with CRC differentiation. Reduced colonic IL-37 and IL-38 is relating to CRC invasion and distant metastasis, suggesting a protective role for IL-38 within the tumor micro-environment. IL-38 is reduced in right-sided CRC compared to left-sided CRC, which is in line with multiple risk factors for right-sided CRC, including the embryonic development of the colon, and genetic differences in CRC between these two sides. Finally, colonic IL-37 and tumor associated neutrophils (TAN) seem to be independent biomarkers of prognostic value, whereas colonic IL-38 seems to be a reliable and independent biomarker in predicting the 5-year survival post-surgery in CRC. However, there is room for improvement in available studies, including the extension of these studies to different regions/countries incorporating different races, evaluation of the role of multi-drug resistance, and different subsets of CRC. It would be useful to determine the kinetics of circulating IL-38 and its relationship with drug resistance/targeted therapy. The measurement of colonic IL-38 at the molecular and cellular level is required to explore the contribution of IL-38 pathways during the development of CRC. These approaches could provide insight for the development of personalized medicine.

## IL-37

IL-37 belongs to the IL-1 superfamily (1) that shares the structural pattern of the IL-1 family (2). IL-37 is a 17–26 kDa protein that corresponds with a gene size of 3.617 kb (3). Constitutive expression of IL-37 has been observed in many leucocytes (NK cells, activated B cells, monocytes), epithelial cells (keratinocytes and epithelial cells), and tissues (e.g., lymph nodes, thymus, lung, intestine, and uterus) (4, 5). IL-37 is mainly considered to be an anti-inflammatory cytokine, because IL-37 is able to suppress innate (2) and adaptive immunity (6), thus explaining the consequent reduction of the host immune response (7), including in tumorigenesis (8). The anti-inflammatory function of IL-37 (7) is partially due to the capacity of IL-37 to inhibit the maturation of dendritic cells (9), and/or to regulate polarization of macrophages, i.e., promoting M1 but inhibiting M2 macrophages (10). Dysregulated IL-37 has been reported in a number of auto-immune diseases, e.g., rheumatoid arthritis (11), psoriasis, Grave's disease and systemic lupus erythematous, and in ulcerative colitis and Crohn's disease (12), and in gestational diabetes mellitus (13), acting perhaps *via* inhibiting the expression and function of pro-inflammatory cytokines (11, 12, 14). In addition, it has been well-reviewed that IL-37 substantially reduces the development of atherosclerosis (15), which has been confirmed in an IL-37 transgenic animal model (16).

## IL-38

IL-38 also belongs to the IL-1 superfamily, which shares ~41% homology with the IL-1Ra and ~43% homology with the IL-36R (17). IL-38 is constitutively expressed in many tissues, e.g., heart, lung, intestine, urogenital system, skin (18), spleen, and tonsils (19). IL-38 is considered to maintain the homeostasis of the microenvironment in these tissues *via* inhibiting/suppressing inflammatory responses (20). The anti-inflammatory role of IL-38 includes the release of IL-38 from apoptotic cells to limit inflammatory macrophage responses (21). An anti-inflammatory role for IL-38 has been demonstrated in a humanized allergic asthma NOD/SCID murine model, showing that IL-38 inhibits proinflammatory cytokines (IL-6, TNF, CCL5, and CXCL10), probably *via* the classical STAT1, STAT3, p38 MAPK, ERK1/2, and NF-κB pathways (22). IL-38 is analogous to the IL-1ab/receptor agonist and IL-1R1, allowing it to mediate its anti-inflammatory activities. In addition, IL-38 plays an important role in maintaining homeostasis by balancing the pro- vs. anti-inflammatory micro-environment (23). Dysregulation of IL-38 can initiate host immunity due to an imbalance of the pro- vs. anti-inflammatory microenvironment, causing inflammatory diseases.

Up-regulated IL-38 expression is detected in inflamed skin (18), the active inflamed tissues of inflammatory bowel disease (24), rheumatoid arthritis (25), psoriatic skin (23), and in drug-induced liver injury patients (26), suggesting the induction of an anti-inflammatory role of IL-38 in response to focal inflammation. On the other hand, IL-38 is downregulated in psoriatic skin in response to stimulation by IL-36γ, IL-17, and IL-22n (all proinflammatory cytokines) (27). This observation suggests that IL-38 counteracts the biological processes induced by pro-inflammatory cytokines in epithelial and endothelial cells, attenuating the severity of autoimmunity. Exogenous IL-38 displays a therapeutic effect *in vivo* in arthritis in an animal model (28).

## Colorectal Cancer

Despite decades of extensive basic and clinical research (29), colorectal cancer (CRC) is still the third leading cause of malignancies (1.93 million cases p.a.) and the second leading cause of cancer-related death (935,000 deaths p.a.) worldwide (30). It is still a major challenge to both clinicians and patients to manage CRC, consequently these patients have poor outcomes and 5-year survival rate (31), since a high proportion of CRC patients are diagnosed at a late stage, exhibiting both deep bowel wall invasion and/or distant metastasis.

A key mechanism in tumorigenesis is an immune response to the tumor, illustrated by the effectiveness of immune checkpoint inhibitors (ICIs) acting against, for example, PD-1, PD-L1, and CTLA-4 (32), because of the evidence that blockade of PD-1 and PD-L1 is an extremely promising approach in cancer immunotherapy for preventing tumor evasion of host tumor antigen-specific T-cell immunity. At the molecular level, CRC can be divided into mismatch repair/microsatellite instability (MMR/MSI) competent tumors (the majority of CRCs) or MMR/MSI incompetent tumors (approximately 4% of CRC) (Hirano 2020). The MMR/MSI incompetent form of CRC is considered to be an “immune-hot” form of CRC, characterized by an increased tumor mutational burden, T cell infiltration, and a substantial anti-tumor immune response within the tumor microenvironment. This form of CRC responds well to ICIs. On the other hand, the majority of CRCs (approximately 96%) are MMR/MSI competent and tumor growth is primarily driven by increased WNT signaling. These tumors are characterized by an immune-exclusive microenvironment, thought to be due to a low tumor mutational burden. Consequently, MMR/MSI competent tumors respond poorly to ICIs, although current trials are underway to increase the inflammatory response within the tumor microenvironment of this form of CRC, using, for example, radiotherapy to induce inflammation and, thus, improve the response of MMR/MSI competent CRCs to ICIs (33). Host immunity plays a critical role in tumorigenesis, especially in CRC, consistent with reports showing that there is a positive correlation between pro-inflammatory mediators and the severity of CRC (34), especially in CRC associated with MMR/MSI incompetent tumors (35).

Clinical evidence demonstrates that there is substantial macrophage infiltration in cancer tissues, termed tumor associated macrophages (TAM). The role of macrophages during the development of cancer is controversial, it is probably related to the terminal differentiation of macrophages, which can be divided into two subsets, based on their surface markers and functionalities, namely classical activated M1 macrophages and alternatively activated M2 macrophages (36). M1 TAM are believed to typically exert anti-tumor functions, including directly mediated cytotoxicity, e.g., the release of ROS and NO, and antibody-dependent cell-mediated cytotoxicity (ADCC) to kill tumor cells; On the other hand, M2 TAM can promote the occurrence and metastasis of tumor cells, inhibit the T cell-mediated anti-tumor immune response, promote tumor angiogenesis, and lead to tumor progression (37). Notably, IL-37 has been shown to inhibit the maturation of M2 macrophages (10).

In the current review, we illustrate the relationship between the expression of IL-37 and IL-38, and colorectal adenocarcinoma, especially for its potential clinical implications, because it is the most common colon and rectum cancer, derived from epithelial cells of the intestinal mucosa (38).

## IL-37 in Colorectal Cancer

Colorectal cancer exhibits substantially reduced IL-37 at both the mRNA and protein levels, compared to that of non-cancer tissues (39). Importantly, a significant correlation is observed between the level of IL-37 expression and disease-free survival, as well as overall survival (39), suggesting that colonic IL-37 provides protection during the development of CRC. Significantly, the level of colonic IL-37 in CRC tissue is inversely correlated with invasion and differentiation (39). It is known that IL-37 is an anti-inflammatory cytokine (2), and consequently inhibits gastrointestinal inflammation following various pathological stimuli, which ultimately suppresses tumorigenesis in the gut *via* inhibiting local inflammation (40). However, any long term chronic stimulation could perturb the balance between a pro- and anti-inflammatory response, resulting in the prolonged persistence of chronic inflammation due to loss of tissue homeostasis (41).

This observation in CRC is partially supported by the findings in gastric cancer, showing that mucosal IL-34 expression is inversely correlated with gastric cancer, including invasion, differentiation, and TNM (42), suggesting that mucosal IL-34 seems to also protect against the development of GC, probably by stimulating the maturation of macrophages via the CSF-1 receptor. The comparison of the observations in CRC and gastric cancer appear contradictory, since IL-37 is an anti-inflammatory cytokine, whereas IL-34 is a pro-inflammatory cytokine, yet these two cytokines both provide a protective role during the development of cancer within the gastrointestinal system. The potential explanation for such a discrepancy is that there is a substantial difference in the microenvironments between the colon and stomach, although these two are both in the gastrointestinal system, e.g., there is a substantial difference in the bacterial flora (>100-fold) between the colon and stomach, which requires different host mucosal regulatory responses for the maintenance of homeostasis (43). Consequently, there are different pathogenesis pathways for gastric cancer and colon cancer, which may involve the induction of different pathways during tumorigenesis. It has been reported that IL-34 is involved in the M-CSF-mediated pathway to regulate recruitment and/or polarization of TAM, maturing into either M1 and M2 subsets (37), with M-CSF polarizing TAM into the immunosuppressive, cancer-promoting M2 phenotype.

The connection between IL-37 and differentiation has been documented in atherosclerotic development. An inverse correlation has been reported between infiltrating M1 cells and the expression of IL-37 in calcified human aortic valves, suggesting that IL-37 suppresses M1 polarization (44). Paradoxically, IL-37 has been reported to promote M1 polarization (7, 9, 10, 44). Such a discrepancy of IL-37 in regulating macrophages between tumorigenesis and atherogenesis may be due to the different systems, i.e., there are a large number of flora in the gut with a great potential to be opportunistic pathogen(s); whereas the cardiovascular system is almost pathogen free. This discrepancy requires further clarification in future.

In addition, it remains to be clarified whether the suppression of IL-37 or IL-34 in CRC and gastric cancer, respectively, results in or from different stimuli, which requires further investigation. A recent study illustrates that exogenous IL-37 inhibits colonic cancer cells and stem cell proliferation and migration *in vitro* and in a CRC animal model *in vivo via* the β-catenin pathway (39). IL-37, belongs to the IL-1 superfamily, and uses the IL-18R and IL-1R8 signaling pathway for regulating its target cells (9), inhibiting dendritic cells. IL-1R8 inactivation is known to be an escape mechanism in CRC (45). However, the precise underlying mechanism involved in such pathways needs further clarification.

Moreover, the right colon is derived from the midgut, while the left side is derived from the hindgut during embryonic development (46). Consequently, there are substantial differences in biological markers, genes and prognosis between left- and right-sided CRC (47). Notably, colonic obstruction from right-sided CRC usually presents at a relatively late stage, compare to that of left-sided CRC (47), due to the more liquid nature of stools and the more capacious nature of the right colon, requiring a more extensive tumor mass to cause obstructive symptoms. However, there is no significant difference in the expression profile of colonic IL-37 between left- and right-sided CRC (39), which may be due to relatively insufficient sensitivity and/or the small sample size, or may be due to a lower protective effect of IL-37 in CRC.

It has been well-established that there is a significant correlation between age and mortality from CRC within a large USA database study (48). To determine whether age is a contributing factor in determining the level of colonic IL-37 expression in CRC patients, a study has been undertaken that compares IL-37 expression between old (>65 year) and young (<65 year) CRC patients. Interestingly, no significant difference is observed in colonic IL-37 levels between old (>65 year) and young (<65 year) CRC patients, suggesting that age is not a contributory factor in determining the level of IL-37 expression. This discrepancy may be due to the majority of the CRC patients studied being close to 65 years, which would reduce the power of age in the study, and/or there may have been an insufficient sample size, particularly from a single medical center (39). Thus, a larger sample size and the use of multiple centers for a study, with different racial background, should be undertaken in a future study. In addition, there is no significant difference in IL-37 levels between males and females, most likely mainly due to most females within the study being post-menopausal, which negates the known benefit of estrogen in reducing the incidence of CRC in women of fertile age (49).

Another research team has shown that IL-37 is localized in the cytoplasm of colonic epithelial cells, but colonic IL-37 expression from CRC tissues is substantially reduced compared to that of non-CRC colonic epithelial cells (50). Colonic IL-37 expression is inversely correlated with the depth of CRC invasion, which is consistent with CRC progression (50). In addition, IL-37 expression is closely related to overall disease-free status and overall survival. Surprisingly, there is no significant correlation between colonic IL-37 expression and differentiation of CRC (50). The data obtained in this study may be related to the sensitivity and specificity of the quantification used in the study, i.e., the IL-37 immunohistochemistry utilized a semi-quantitative, semi-subjective methodology using simple visual inspection, which might compromise objective evaluation. Ideally, an immunohistochemical staining quantification using an automated computerized system, e.g., ImagePro Plus, should be used for more objective and accurate evaluation (42).

The role of tumor associated neutrophils (TAN) is a rather novel concept, suggesting that TANs can promote development of tumors *via* enhancing inflammation and subsequent angiogenesis and extracellular matrix remodeling and metastasis (51). It has been demonstrated that a combination of IL-37 and TANs seems to be a reliable prognostic biomarker for CRC, using multivariate analysis (50). The finding suggests that IL-37 also reduces inflammation in the microenvironment by inhibiting recruitment of neutrophils, which is well-known as an acute and/or chronic source of inflammation (50).

The protective role of IL-37 in tumors has been demonstrated in a number of cancers, e.g. in human hepatocellular carcinoma (52), perhaps *via* inhibiting M2 macrophages (10), in lung cancer (53) *via* inhibiting angiogenesis, and in an animal model (54). Furthermore, a high level of IL-37 has been shown to correlate with increased CD1a^+^ dendritic cell infiltration, and correlates well with the overall survival rate in hepatocellular carcinoma (8). The consequence of increased dendritic cells in anti-tumor immunity perhaps involves enhanced professional antigen presentation with subsequent increased differential polarization of macrophages (8). This could explain the differential role of macrophages during the development of malignancies, which may exhibit different mechanisms of carcinogenesis or micro-environments.

## IL-38 and CRC

IL-38 is constitutively expressed in very low amounts in many tissues and has an anti-inflammatory role (20) for maintaining homeostasis within the micro-environment. The anti-inflammatory role of IL-38 includes the release of IL-38 from apoptotic cells to limit inflammatory macrophage responses (21). Thus, any disturbance of IL-38 can cause maladaptive clinical responses. From a functionality point of view, IL-38 displaces inflammatory signaling, analogous to the IL-1ab/receptor agonist and IL-1R1, as an anti-inflammatory mediator.

Chen et al. found that colonic IL-38 production in CRC is mainly located in the cytoplasm of epithelial cells in non-CRC colonic tissue, but is substantially down-regulated within CRC colonic tissue compared to that of matched non-CRC colonic tissue (55), suggesting that IL-38 is involved in the development of CRC. This is supported by the finding that an inverse correlation is also detected between colonic IL-38 production and the extent of differentiation of CRC, suggesting a protective role of colonic IL-38 to maintain normal homeostasis of the intestinal mucosa within the micro-environment. The protective role of IL-38 in CRC is in line with other studies, showing that IL-38 provides protection against gestational diabetes mellitus-induced inflammation within the placenta (56), and in the context of the demonstration of a close correlation between inflammatory bowel disease and the development of CRC (57).

As stated above, there are physiological and anatomic differences between the right and left sides of the colon (46), arising from embryonic development, which contributes to different clinical symptoms for left- and right-sided CRC patients, resulting in a later diagnosis of right-sided colon cancers (47). Colonic IL-38 expression has been shown to be nearly half the level in right-sided CRC compared to that of the left side (55). The precise underlying mechanism is unclear, but it may be related to the differentiation and/or extent of invasion of CRC, correlating to clinical symptoms during the development of CRC. In addition, defective MMR/MSI positive CRC is more frequent in right-sided CRC and is associated with a higher level of inflammation within the tumor microenvironment. Thus, low levels of IL-38 may be a complex reflection of this higher level of inflammation. The relationship between MSI and IL-38 expression has not yet been investigated.

These data also correlate with the finding that reduced colonic IL-38 is detected in larger CRCs with a tumor size >5 cm, compared to that of ≤5 cm (55). Notably, right-sided CRC is frequently diagnosed at a later stage, with the tumor size being larger, due to the capacious nature of the proximal colon (47). It is well known that the tumor size and depth of invasion (58) correlates with prognosis and overall survival rate of primary CRC patients (59).

As stated above, age is an important factor during the development of CRC (48), however, no significant difference of colonic IL-38 is detected between young and old (cut-off at 70 years) CRC patients. This may be due to the majority of the CRC patients within the study being within a relatively old group, close to 65 years, which may dilute the power of the statistics, and/or the sample size being relatively small and from a single center (55). Similarly, no significant difference in CRC IL-38 expression is observed between males and females, which is consistent with similar findings for colonic IL-37 expression in CRC tissues between males and females (39, 50), and is probably due to the majority of these CRC female patients being postmenopausal, consequently having already lost the protective power of estrogen (49).

Although the precise underlying mechanism involved in the reduction of colonic IL-38 during the development of CRC is unclear, it is speculated that compromised intestinal mucosal immunity might contribute to the disturbance of the balance between pro- and anti-inflammatory cytokines in the colon (27) within the susceptible cohort (60). Consequently, the resultant dysregulated inflammation within the intestinal mucosal immunity of these individuals (61) is eventually conducive to the development of CRC (34, 62). Furthermore, the finding that IL-38 is also inversely correlated with TNM classification in CRC (55), suggests that reduced local and, probably, circulating IL-38 might promote malignant cell short-distance invasion or long-distance metastasis. This is further supported by the findings that there is a close correlation between chronic inflammation and the development of gastrointestinal cancer (63).

In contrast, a correlation has been reported between high IL-38 expression and poor differentiation of lung adenocarcinoma (64). Moreover, within this lung cancer cohort, high IL-38 expression also positively correlated with high TNM and with a shorter disease-free survival, but only in PD-L1 negative tumors where T cell activity is presumably not suppressed. These data suggest that IL-38 promotes the development of lung carcinoma, with an inverse correlation with PD-1/PD-L1 expression, rather than inhibits the development of lung cancer. The precise underlying mechanism/s explaining the apparently contradictory involvement of IL-38 in CRC compared to lung cancer is still unclear. We speculate that the discrepancy in IL-38 expression in CRC vs. non-small cell carcinoma of the lung may be due to different immune system responses within the two tissues, which experience quite different microflora loads and/or microenvironments, with the differing stimuli tipping the different host responses in opposite directions, which requires further clarification.

It is well-known that the IL-36 isoforms are pro-inflammatory cytokines, which contribute to activation and proliferation of leucocytes, including macrophages, dendritic cells, and lymphocytes (65). It has been demonstrated that there are differential roles for IL-36α, β, and γ in CRC (66), when the level of expression of these interleukins is evaluated for their capacity to predict 5-year survival among CRC patients. Considering that the pathophysiological function of IL-38 is to bind *via* the IL-36 receptor to block IL-36 signaling (20, 67), these data are consistent with the observed correlation between high expression of IL-38 and improved 5-year survival in CRC (55). The direct interaction between IL-38 and IL-36 during the development of CRC remains to be explored. Our preliminary observations have shown the induction of larger and more numerous CRCs in IL-38 GKO animals compared to the wild-type, supporting speculation that IL-38 plays a critical protective role during the development of CRC, perhaps *via* regulating host immunity. Although the counteractive role between IL-38 (55) and IL-36s (66) in the development of CRC remains to be confirmed, considering the known anti-inflammatory role of IL-38 and pro-inflammatory role of the IL-36 isoforms, this mechanism likely contributes to the dysregulated intestinal immunity that occurs during CRC pathogenesis.

In conclusion, this mini review makes the case that there are some similarities between IL-37 and IL-38 in inhibiting tumorigenesis of CRC, yet these two interleukins use different signaling pathways in mediating and/or interacting with cancer cells in the tumor microenvironment. IL-37 and IL-38 are reliable prediction factors for determining the prognosis of CRC, and constitute strong and reliable predictors of survival post-surgery. There is room for improvement in these supporting data, including the extension of the study to different regions/countries evaluating different races, determining the effects of multi-drug resistance, and clarifying expression levels in morphological and molecular subsets of CRC. It would be useful to determine the kinetics of circulating IL-38 and its relationship with drug resistance/targeted therapy. The measurement of colonic IL-38 at both the molecular and cellular level is required to explore the contribution of IL-38 pathways during the development of CRC. Furthermore, these data also suggest that IL-37 and IL-38 may serve as therapeutic targets in the post-surgery management of CRC patients, particularly in the development of personalized precision medicine.

## Author Contributions

JD, ZH, XC, JF, DH, BH, XL, and SB conceptualized the idea and contributed to writing the article. All authors contributed to the article and approved the submitted version.

## Conflict of Interest

The authors declare that the research was conducted in the absence of any commercial or financial relationships that could be construed as a potential conflict of interest.

## Publisher's Note

All claims expressed in this article are solely those of the authors and do not necessarily represent those of their affiliated organizations, or those of the publisher, the editors and the reviewers. Any product that may be evaluated in this article, or claim that may be made by its manufacturer, is not guaranteed or endorsed by the publisher.
